# Mutations in the *Arabidopsis RPK1* gene uncouple cotyledon anlagen and primordia by modulating epidermal cell shape and polarity

**DOI:** 10.1242/bio.20135991

**Published:** 2013-08-22

**Authors:** Miriam Luichtl, Birgit S. Fiesselmann, Michaela Matthes, Xiaomeng Yang, Ottilie Peis, Andrä Brunner, Ramon A. Torres-Ruiz

**Affiliations:** Lehrstuhl für Genetik, Technische Universität München, Wissenschaftszentrum Weihenstephan, Emil-Ramann-Strasse 8, D-85354 Freising, Germany

**Keywords:** Angiosperm evolution, *Arabidopsis*, Cotyledon, Embryo development, *RPK1*

## Abstract

Plant seedlings have either one or two cotyledons. The mechanisms that regulate this organ number are poorly understood. Mutations in the *RECEPTOR-LIKE PROTEIN KINASE1* (*RPK1*) gene of the dicot *Arabidopsis* have only one cotyledon, with low penetrance due to complex genetic redundancy. An analysis of patterning genes required for cotyledon initiation showed that these have normal expression patterns, defining the cotyledon anlagen, in *rpk1*. This was also true for key genes, which organize the shoot apical meristem (SAM). By contrast, epidermal cell shape and polarity were compromised in *rpk1* embryos, as evidenced by disturbed polarity of the auxin efflux carrier PIN1. PIN1 is required for the establishment of auxin maxima, which induce and maintain organ primordia. The effects in *rpk1* mutants manifest in a spatially and timely stochastic fashion probably due to redundancy of *RPK1*-like functions. Consistently, auxin maxima showed a stochastic distribution in *rpk1* embryos, being at times entirely absent and at other times supernumerary. This variability may explain how monocotyledonous seedlings and cotyledon shape variants can developmentally arise in *Arabidopsis* and possibly in other plants.

## Introduction

The determination of organ number is specifically controlled in all organisms. Plant seedlings, for example, may have either one or two cotyledons, depending on the species. The determination of cotyledon number is a critical process during embryogenesis. Its importance is reflected in modern taxonomy, which recognizes eudicots with two and monocots with one cotyledon, as monophyletic groups (Crane et al., 1994). Exceptions from normal cotyledon number in angiosperms are known in several genera (e.g. Cronquist, 1988). Mutants affecting this trait have been characterised. For instance, some mutants in *Antirrhinum* develop higher cotyledon numbers with incomplete penetrance, which can in part be enhanced or suppressed by additional modifier genes depending on the genetic background (Stubbe, 1963). In *Arabidopsis* the apical polarity of the auxin transporter PIN1 is essential for cotyledon development (Benková et al., 2003; Friml et al., 2004). Mutants interfering with this process alter cotyledon number. For instance, mutants of the AGC kinase *PINOID* (*PID*) and D-myo-inositol-3-phosphate synthase (MIPS) frequently produce abnormal supernumerary cotyledon numbers (Bennett et al., 1995; Luo et al., 2011). In contrast, combination of *pinoid* with mutants of related kinases, auxin-synthesis genes, the *NPH3*-like gene *ENHANCER OF PINOID* (*ENP*) and *PINFORMED1* (*PIN1*) itself result in cotyledon-less seedlings with variable penetrance up to 100% (Furutani et al., 2004; Treml et al., 2005; Furutani et al., 2007; Cheng et al., 2008; Dhonukshe et al., 2010; Won et al., 2011).

Interestingly, mutants specifically segregating a monocotyledonous phenotype are rare but are known from pea (*sic*; Liu et al., 1999) and from mutations in the *Arabidopsis* Ser/Thr kinase *RPK1* with low penetrance (4.8%) (Nodine and Tax, 2008). Assuming that this low penetrance might be due to gene redundancy, double mutants of *RECEPTOR-LIKE PROTEIN KINASE1* (*RPK1*) and the related *RPK2/TOAD2* were analysed (Nodine et al., 2007; Nodine and Tax, 2008). This combination resulted in higher frequencies of the monocotyledonous phenotype but also in other severe pattern effects as well as a high incidence of embryo lethality. The severity of these defects also became visible in the remaining embryos, which in some regions even failed to express key genes like *SHOOT MERISTEM-LESS* (*STM*), *AINTEGUMENTA* (*ANT*) and *PIN1* (“defective half” and “toadstool” embryos) (Nodine and Tax, 2008). This work demonstrated a crucial role of these Receptor Like Kinases (RLKs) for embryo radial patterning notably the protoderm (Nodine et al., 2007; Nodine and Tax, 2008).

Although a number of mutants affecting cotyledon number have been identified, the mechanisms that regulate this organ number in *Arabidopsis*, in particular the decision between two versus one cotyledon, are poorly understood. *RPK1* and *RPK2/TOAD2* appear to be key players in this process. The pleiotropic phenotypes of *rpk1 rpk2/toad2* double mutants, however, have precluded an analysis of their function in radial patterning versus in the specific control of cotyledon number. In order to identify the cotyledon specific function of *RPK1*, we analysed *rpk1/rpk1* single mutant embryos, which exclusively affect cotyledon development in the embryo. In this study, we used a new strong *rpk1* fast neutron allele (*rpk1-7*), which alleviated but did not eliminate the penetrance problem. We show that in *rpk1* cotyledon anlagen are perfectly established. The expression of organisers and key regulators of the shoot apical meristem (SAM) and cotyledon development are essentially unaffected. However, on a cellular level, *rpk1* specifically but stochastically interferes with cell division and PIN1 polarity. Both effects are followed by the stochastic alteration of position and number of auxin maxima leading to one absent and one sometimes abnormally shaped cotyledon. Thus, in contrast to the aforementioned *rpk1 rpk2/toad2* double mutant study, monocotyledonous seedlings arise from a specific cellular defect in an almost normal embryo. The identification of this early effect assigns a function to RPK1 in stabilising auxin maxima. Although this finding explains how numerous cotyledon variants can arise in an otherwise dicot embryo in *Arabidopsis*, it could have evolutionary implications for plants in general.

## Results

### The mutant *abanico^FN9-3^* represents a strong *RPK1* allele (*rpk1-7*) in Columbia ecotype background

For convenience, the terms monocot and dicot embryos/phenotypes are used in the following. This should not be confused with the taxonomic meaning of these terms.

**Fig. 1.**
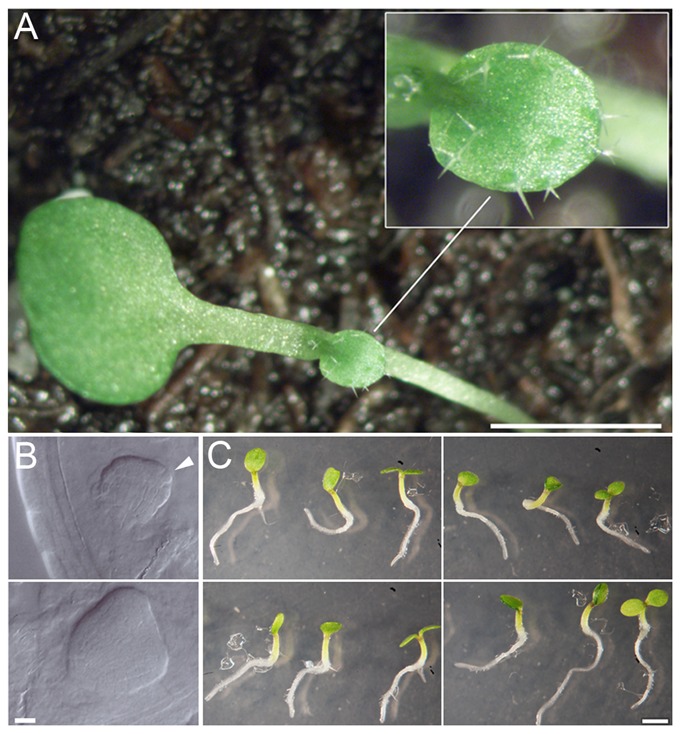
The *rpk1-7* allele phenotype. (A) Monocotyledonous seedling phenotype of the *rpk1-7* allele (back-crossed to wt), inset shows the primary leaf with trichomes. (B) *rpk1-7* embryos from globular to heart stage (arrowhead points to enlarged epidermal cells). (C) Homo- and transheterozygous *rpk1* seedlings. Top left: *rpk1-6/rpk1-6*; top right: *rpk1-7/rpk1-7*, bottom: *rpk1-7/rpk1-6* from reciprocal crosses (right and left, respectively). Scale bars: 1 cm (A); 10 µm (B); 1 mm (C).

**Fig. 2.**
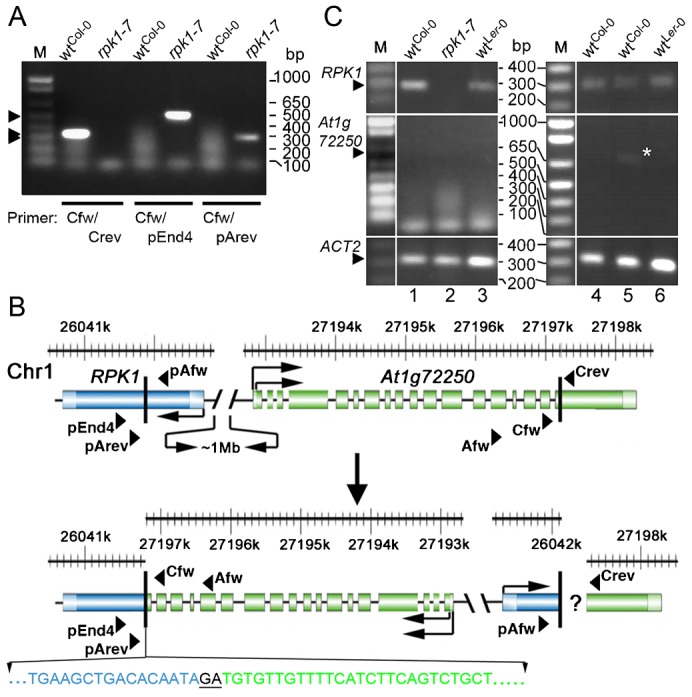
Molecular characterization of *rpk1-7*. (A) Amplification of fragments crossing the fusion point between *RPK1* and *At1g72250*. Primer pair Cfw/Crev amplifies an *At1g72250* fragment in Col-0 but not in *rpk1-7* while Cfw/pEnd4 and Cfw/pArev produce fragments in *rpk1-7* but not in Col-0, respectively. (B) Structure of wild-type (top) and the fast neutron induced rearrangement in *rpk1-7* (bottom); positions of exons/introns and primers are indicated. The sequence across the left fusion point is given (blue: *RPK1*; green: *At1g72250* and black and underlined: shared sequence). The position but not the precise arrangement of the right *RPK1*/*At1g72250* fusion point is known. (C) Expression analyses of *RPK1*, *At1g72250* and *ACT2* (control) in *rpk1-7*, Col-0 and L*er*-0 of leaves (1, 2, 3), embryos (4) and seedlings (5, 6), respectively. Note the weak *At1g72250* band in Col-0 but not L*er*-0 seedlings (star). Arrowheads in panels A and C indicate the size of expected bands.

In a fast neutron screen we found a line named *abanico^FN9-3^* (*aco*, “abanico” spanish “fan”) segregating monocot seedlings with a penetrance of 8.38% (S_n−1_ = +3.93%; Fig. 1A,B). In rare cases, the monocot seedlings developed no or a late SAM. Other seedlings developed irregularly lobed, unequally sized ( = anisocot) or spatially displaced cotyledons (supplementary material Fig. S1). In the original line the total penetrance of cotyledon defects in seedlings was 10.53% (S_n−1_ = +4.44%). This frequency could be altered by crossing *abanico^FN9-3^* with different ecotypes (supplementary material Fig. S2). All seedlings, except those without SAM, developed to fertile plants. Molecular mapping located *aco^FN9-3^* near the marker *ap1* on the lower arm of chromosome 1 (Fig. 2; supplementary material Fig. S3). Based on published descriptions, different candidate genes in this interval were tested by PCR analysis (see Materials and Methods). An improved TAIL-PCR method (Liu and Chen, 2007) identified break points in the genes *RPK1*/*At1g69270* and *At1g72250* (a glucose-binding kinesine homolog) (Vanstraelen et al., 2006), respectively, indicating a possible inversion (Fig. 2). Specific primer combinations could bridge one fusion point between the C-terminus of *RPK1* and the N-terminus of *At1g72250* (Fig. 2A,B). Other primers amplified parts of the N-terminus of *RPK1* (or *At1g72250*) but did not bridge the second fusion point, which probably hints to a more complex rearrangement on this side.

In contrast to analyses in wild-type, expression analyses showed no detectable transcript for *RPK1* in the mutant (Fig. 2C). Therefore, the isolated line was considered to be at least a null-allele of *RPK1*. Interestingly, *abanico^FN9-3^* exhibited a higher monocot penetrance than the reported *rpk1* null-allele (Nodine and Tax, 2008) and no other seedling phenotype. We therefore asked whether the *At1g72250*-mutation could be a modifier (enhancer) of *rpk1* in *abanico^FN9-3^*. Although the function of *At1g72250* is not known, homology places it in the area of cell division and chromosome movement (Vanstraelen et al., 2006). However, *At1g72250* is not an enhancer of *rpk1* because firstly, it was not expressed in embryos and only weakly if at all in later stages (Fig. 2C). Secondly, *aco^FN9-3^* only displayed the cotyledon phenotype known from *rpk1* (Fig. 1). Thirdly, available T-DNA insertion lines of *At1g72250* (supplementary material Fig. S3) did not display any conspicuous seedling phenotype. Fourthly, trans-heterozygotic seedlings of *aco^FN9-3^* and the allele *rpk1-6* had the same phenotype as *aco^FN9-3^/aco^FN9-3^* and *rpk1-6/rpk1-6* seedlings (Fig. 1C). Fifthly, transgenic *RPK1p:RPK1::GFP* in *aco^FN9-3^* lowered the frequency of the monocot phenotype in the F2, indicating a rescue effect (1.88±3.1%; *n* = 19 plants with 234 counted seedlings on average). The reduction of the mutant phenotype frequency by 75%, i.e. from 10.53% (S_n−1_ = +4.44%) to 1.88% (S_n−1_ = ±3.1%) indicates that the *RPK1* transgene largely if not completely rescues the phenotype. The remaining 1.88% monocots likely represent the 25% F2 plants with monocot phenotype, which do not carry a transgene. We conclude that the breakpoint in *RPK1* but not in *At1g72250* affects embryo development in *aco^FN9-3^* and renamed this mutant *rpk1-7* (for overview of known alleles see supplementary material Table S1). The same experiment also minimizes a possible impact of genes within the complex rearrangement on the right border of the inversion, if there is at all an influence of this region on the phenotype. This conclusion is fostered by the identical phenotypes of the homo- and trans-heterozygotic seedlings mentioned above.

The monocot penetrance in *rpk1-7* can be significantly altered in diverse ecotype backgrounds (supplementary material Fig. S2) as well as in some crosses with different marker/mapping lines (not shown). In order to maintain an isogenic genetic background, we therefore excluded plants of mixed origin because these might harbour variable numbers of modifiers. Taking advantage of the monocot penetrance frequency we predominantly, but not exclusively, used the *rpk1-7* allele for further analysis.

### The expression of SAM-specific genes remains normal in monocot *rpk1* embryos

We did not detect expression pattern differences of the genes analysed between wild-type dicot and *rpk1-7* dicot embryos. Therefore, dicots from both backgrounds served as reference for monocot *rpk1-7* embryos.

**Fig. 3.**
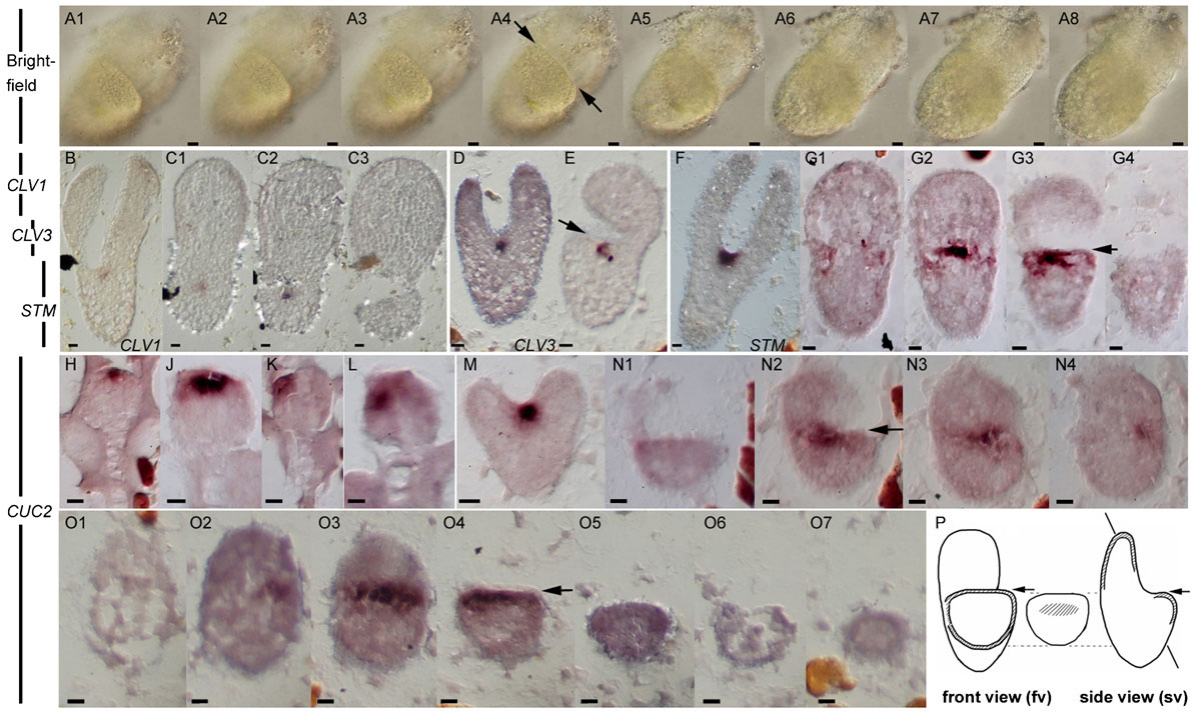
In situ hybridisation of *rpk1-7* monocot and dicot embryos with representative genes organising the SAM. Bright field and in situ analyses with the gene probes are indicated. (A1–A8) Series of optical sections of a monocot embryo in front view (fv; see also P for orientation). (B–C3) *CLV1* expression (panels C1–C3 are oblique sections). (D,E) *CLV3* expression. (F–G4) *STM* expression. (H–O7) *CUC2* expression. Note the differences between the front views (fv) and the side views (sv). (B,D,F,M) Dicot heart and torpedo stage embryos, respectively. (H–L) Globular embryos. All other figures show monocot heart stage embryos. (P) Scheme of side view (sv) and front view (fv) sections in monocot embryos (lines indicate orientation of oblique sections in panels C1,C2). Arrows point to the plateau of the apex. For further details see text. Scale bars: 10 µm.

Frontal sections gave a characteristic series of cross-sections through the cotyledon-less half of the embryo (e.g. Fig. 3A1–A8). *CLAVATA1* and *3* (*CLV1*, *CLV3*) were selected as representatives of genes involved in SAM stem cell identity (Fletcher et al., 1999). Their expression is known to lie in the centre of the shoot apical meristem in between the cotyledons and embraces few cells (Fig. 3B–E). The in situ hybridization showed essentially the same expression pattern for *CLV1* (Fig. 3B,C1–C3) and *CLV3* (Fig. 3D,E) in *rpk1-7* dicot and monocot embryos, respectively. The SAM expression of *CLV1* was also shown with the construct *CLV1p::mGFP-ER5* (Gallois et al., 2002) introduced into *rpk1-7* (supplementary material Fig. S4). Next we studied the expression pattern of the meristem-specific gene *STM*. In wild-type/*rpk1-7* dicot embryos, *STM* adopts a significantly broader expression domain (Long et al., 1996) than the *CLV* genes because it also functions to suppress differentiation of cells in the immediate proximity of stem cells (Fig. 3F–G4). Series of monocot embryo sections showed *STM* expression in an analogous position to dicot embryos (compare Fig. 3F with Fig. 3G1–G4). Since the second cotyledon primordium was missing, *STM* signal was found in cells of the apex forming a plateau adjacent to the single cotyledon. Note that the *STM* domain did not extend to the border of the apex plateau where the missing cotyledon primordium would be expected to arise (Fig. 3G4). Finally, we tested the expression of *CUP-SHAPED COTYLEDON2* (*CUC2*), which redundantly controls organ separation (Aida et al., 1999). Consequently, its expression in the dicot embryo appeared between the bases of cotyledons laterally to the SAM (Fig. 3H–O7). The *CUC2* expression domain was visible from early on in the centre of globular *rpk1-7* embryo stages as in wild-type (Fig. 3H,J). In few cases, this domain appeared laterally shifted from the centre of the apex (Fig. 3K,L), possibly representing presumptive monocot embryos. Thus as in dicots, *CUC2* adopts an expression domain in order to suppress the fusion of the margins of the remaining cotyledon primordium. In monocot heart stages, *CUC2* displayed as in wild-type an expression pattern similar but not identical to *STM*.

### The expression of cotyledon/organ specific genes define the cotyledon anlagen in wild-type and *rpk1* embryos

**Fig. 4.**
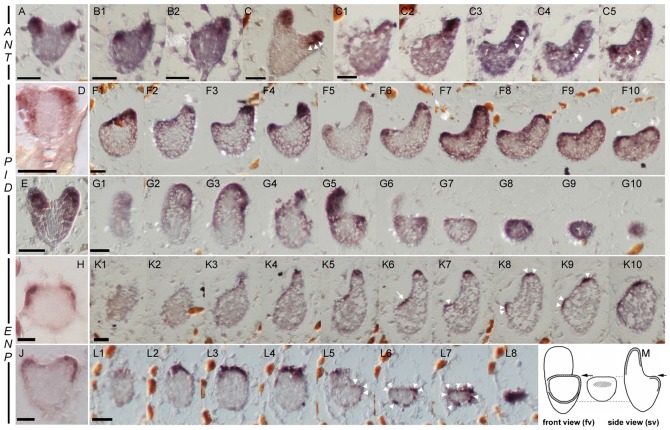
In situ hybridisation of *rpk1-7* embryos with cotyledon specific genes. Shown are transition stage embryos (D,H), heart stage dicot embryos (A,C,E,J) and monocot *rpk1-7* embryos (all others). Gene probes used for in situ analyses are indicated on the left. All figures show heart stage embryos except panels D and H, which show transition stage embryos. (M) Overview scheme of side view (sv) and front view (fv) sections in monocot embryos. Note the strong *ANT* hybridisation signals in A–C5 in the tips of the cotyledon primordia as well as in vascular precursors in panels C and C3–C5, respectively (white arrowheads) and the broad *PID* hybridisation signals in contrast to the *ENP* hybridisation signals restricted to the epidermis. The white arrow (in K6) points to a weak *ENP* expression signal in the region of the SAM. In monocot embryos, all three genes show a clear expression in the cotyledon primordium as well as in the cotyledon anlage for the second primordium, which had not produced a cotyledon primordium. Scale bars (given in the first panel for a series): 30 µm.

We further tested organ specific genes as for instance *AINTEGUMENTA* (*ANT*) whose expression concentrates in the central tissue of cotyledon primordia extending into the hypocotyl and excludes the region of the SAM (Elliott et al., 1996; Treml et al., 2005). Surprisingly, *ANT* displayed its known pattern in dicot as well as in monocot embryos (Fig. 4A–C5). As in wild-type, *ANT* was not expressed in the centre of the monocot apex (compare Fig. 4A with Fig. 4B1, and Fig. 4C with Fig. 4C3–C5). Most notably, *ANT* expression was significant at the border of the apex, where the second cotyledon primordium was missing (Fig. 4B1,C5).

*ANT* is a valuable expression marker for incipient cotyledon primordia but *ant* mutants do not have a cotyledon phenotype (Elliott et al., 1996). Therefore we analysed the genes *ENP* and *PID*, which play a crucial role in the initiation and maintenance of cotyledons (Treml et al., 2005; Furutani et al., 2007). In wild-type, *PID* and *ENP* are known to be expressed at the flanks of globular and transition stage embryos where the presumptive cotyledon primordia are expected (Fig. 4) (Furutani et al., 2007; Christensen et al., 2000). This expression concentrates to the tips of cotyledon primordia in embryo heart stage (Fig. 4E,J). *PID* displays a broader expression domain including epidermal and sub-epidermal cells down to the hypocotyl (Fig. 4D,E) while *ENP* adopts a more restricted expression domain in the epidermal cells with weak signals in the hypocotyl and in the SAM region (Fig. 4H,J) (Furutani et al., 2007). Detailed analyses of *PID* and *ENP* expression in serial sections of heart stage monocot embryos displayed clear signals for *PID* in the tip of the growing cotyledon but also at the site where the second cotyledon should normally grow (Fig. 4F1–F10, side view, Fig. 4G1–G10, front view). Similarly, *ENP* expression was found in the tip of the normally grown cotyledon and at the region where the second cotyledon should develop (Fig. 4K1–K10, side view, Fig. 4L1–L8, front view). Notably, *PID* expression extended to sub-epidermal cells while *ENP* remained in the epidermis even in the region of the missing or reduced cotyledon (compare Fig. 4F4 with Fig. 4K8,K9, and Fig. 4G8,G9 with Fig. 4L6,L7). Both expression patterns were exactly identical to wild-type. Cases with a cotyledon stump (rather than a completely missing primordium) indicated a strongly retarded growth of one primordium in an anisocot embryo (Fig. 4F1–F10). Taken together, expression of *PID* and *ENP* are not disturbed in *rpk1* embryos. Conversely, *RPK1* expression was not suppressed in the cotyledon-less double mutant *pid enp* (supplementary material Fig. S5).

### The orientation of cell walls and the cellular polarity of PIN1 are sometimes altered in the *rpk1* epidermis of incipient cotyledon primordia

**Fig. 5.**
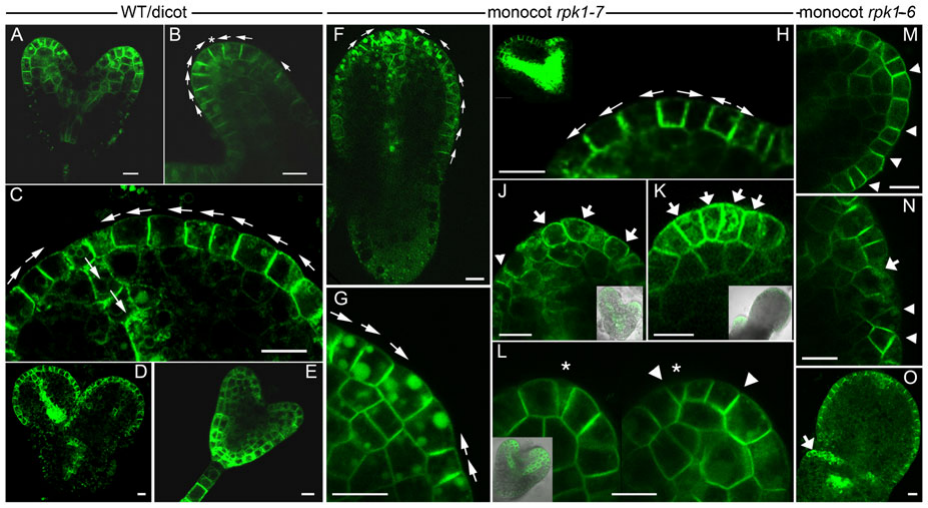
Localisation of PIN1:eGFP in *rpk1-6* and *rpk1-7* embryos, respectively. Dicot/wild-type (A–E) and aniso/monocot embryos of *rpk1-7* (F–L) and *rpk1-6* (M–O) embryos, respectively, are indicated. (A–D) The regularity of wild-type cell sizes and PIN1:eGFP polarity in the epidermis as well as in a terminal cell (at a convergence point) and sub-epidermal vascular precursor cells (panel C is a magnification of panel D). (E) The distribution of RPK1:GFP in a heart stage embryo. (F,G) A torpedo stage monocot embryo with a relatively regular epidermal layer (panel G is a magnification of panel F). (H–O) The variability of cell proportions, cell wall orientations and PIN1:eGFP distributions in different *rpk1* specimen with cotyledon defects (panels H–L are magnifications of embryos in insets). Arrowheads point to cell walls, which are oblique or which separate cells of different size. Variable size proportions and cell wall orientations can be seen in panels J and L–N. Abnormal PIN1:eGFP concentrations and distributions (i.e. bi- and multipolar, “u/n-shaped”) can be seen in panels H–K, N and O (broad arrows). Note that some strong fluorescence in figures showing a complete embryo results from the cumulative fluorescence of many cells in the focus chosen. Additional symbols: small arrows highlight orientation of PIN1:eGFP and stars indicate terminal cells in the convergence points where recognizable. Scale bars: 10 µm.

**Fig. 6.**
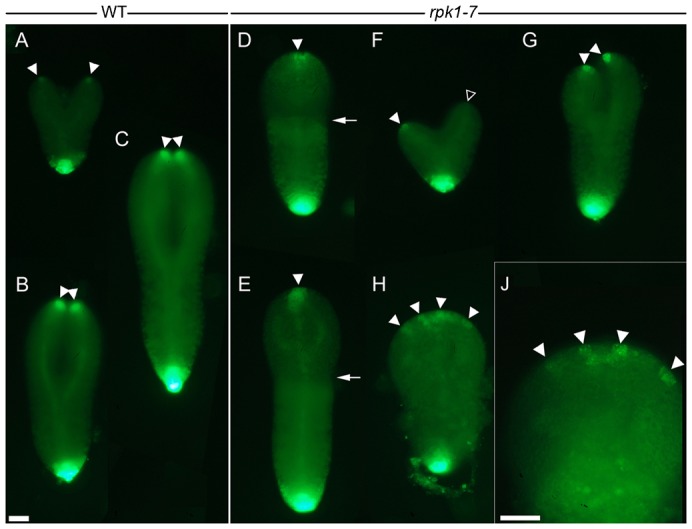
Number and distribution of cotyledon auxin maxima in wild-type and *rpk1-7*. Wild-type and *rpk1-7* monocot and anisocot embryos are indicated. (A–C) The wild-type localisation of auxin maxima (arrowheads) in developing cotyledons of late heart (A), early torpedo (B) and mid torpedo (C) stage dicot embryos. (D–J) The number and position of auxin maxima in monocot (D,E,H,J) and anisocot (F,G) *rpk1-7* embryos of comparable stages. (J) A magnification of panel H. Arrows indicate the plateau of the embryo apex in monocots. A white triangle vs an arrowhead point to auxin maxima of different intensity in an anisocot (F). (H,J) A monocot embryo with four auxin maxima (arrowheads). Scale bars: 30 µm (A–H), 30 µm (J).

PIN1 has a key role as auxin transporter in the apical region of the embryo and is localised on the apical side of epidermal cells oriented towards the tip of the emerging cotyledon primordia (Fig. 5) (Benková et al., 2003). The polar auxin flux leads to an auxin maximum at each primordium (Fig. 6), which establishes and supports its growth in wild-type (Benková et al., 2003). Furthermore, epidermal cells display a regular shape with comparable sizes between each other and the cell walls are perpendicular to the surface (Fig. 5). The convergence point (Scarpella et al., 2006) at the tip of the primordium contains cells with PIN1 polarity pointing to at least one terminal cell. This displays a PIN1 orientation towards the sub-epidermis (Fig. 5A–D). Vascular precursor cells in the sub-epidermal tissue possess significant PIN1 protein concentrations and display a basal and lateral localisation such that auxin is guided towards the hypocotyl and root tip, respectively (Fig. 5C). This PIN1 pattern was mostly not altered in aniso- and monocot *rpk1-6* and *rpk1-7* embryos. For instance, in torpedo stage monocot embryos PIN1 arrangement can be almost wild-type (compare Fig. 5A–D with Fig. 5F,G). However, a detailed analysis revealed rare but detectable deviations in altered as well as in normal cotyledon primordia.

Firstly, epidermal cells had sometimes different or altered size proportions (Fig. 5J,L–N). Secondly, oblique cell walls were found alongside with reduced cell sizes (Fig. 5L–N). Thirdly, some cells displayed weak or an almost global or multipolar (“u- or n-shaped”) distribution of PIN1 in the membrane, which sometimes included a localisation towards the endosperm space, respectively (Fig. 5H–K,N,O). Although these alterations were prominent at the tip, they were also found in more lateral primordia positions. This and their absence/occurrence in embryos of different developmental stages, indicated that these alterations occur spatially and timely in a stochastic fashion during embryogenesis.

The question is how frequently erroneous cell shapes and polarities occur during the development of an *rpk1* embryo. During our analyses, we noticed that cotyledon defects (mostly anisocot, i.e. cotyledons with unequal size) were more abundantly represented in embryos than in seedlings. Since it is almost impossible to reliably assess numbers of abnormal vs normal epidermal cells within embryos having hundreds or thousands of cells, we counted how frequently abnormal embryos occurred. In fact, the abundance of abnormal embryos (slightly) different to wild-types was astonishingly high. In five different plants we counted up to 50% (one case 64%) such embryos from globular stage onwards (supplementary material Table S2). Considering the penetrance found in seedling stage, this shows that weak anisocots compensate with extended proliferation during late embryogenesis to give almost normal dicot seedlings.

Finally, it should be noted that RPK1 protein expression overlaps with PIN1 (compare Fig. 5D with Fig. 5E). RPK1 shows an epidermal as well as a (weak) sub-epidermal tissue localisation (Fig. 5E) (Nodine et al., 2007). Interestingly, its abundance is high in hypocotyls and root tip regions and low in the apical regions.

### The number and position of auxin maxima varies in cotyledons of *rpk1* mutants

Next we analysed auxin maxima in *rpk1*-7 homozygous plants carrying the construct with the synthetic auxin responsive promoter DR5 preceding the GFP gene (Benková et al., 2003). In the F3 progeny we inspected dicot and cotyledon-defect embryos up to mid-torpedo stages (Fig. 6). Dicot *rpk1-7/rpk1-7* embryos displayed auxin maxima at their tips, which were not different to wild-type maxima. The maxima in the mutant can be weak in heart stages and become stronger during further development, but as in wild-type never reach the intensity of the root-tip maximum (Fig. 6). The different cotyledon-defect embryos displayed different variants of auxin maxima arrangement. Monocots with a lean-shaped cotyledon (in its dimensions similar to wild-type cotyledons) had a single, terminal auxin maximum (Fig. 6D,E). Anisocot embryos mostly displayed clear maxima at the tips of their cotyledon primordia (Fig. 6G). Sometimes the strength of the maxima was significantly different and the weaker of the two could be in the larger cotyledon (Fig. 6F). Another interesting pattern was displayed in monocot embryos with relatively broad or large cotyledon primordia harbouring several maxima evenly distributed along the margin of the primordium (Fig. 6H,J; supplementary material Fig. S6). Accordingly, we found seedlings with two or more auxin maxima correlating with an altered cotyledon shape (e.g. looking like two fused cotyledons; supplementary material Fig. S6). The stochastic *rpk1* defects also explained the occurrence of dicot seedlings with one normal and one irregular cotyledon (supplementary material Fig. S1). Taken together, the pattern of growth promoting auxin maxima matched with the number and shapes of cotyledon variants.

## Discussion

The specific generation of monocotyledonous seedlings is a rare trait among mutants affecting cotyledon number. This study shows that *RPK1* appears to be a key gene regulating cotyledon number. Mutations in *RPK1* do not affect expression of key genes in cotyledon anlagen but they compromise epidermal cell shape and polarity in cotyledon primordia. This alters the number and position of auxin maxima and in turn the number and shape of cotyledons. This event is infrequent and stochastic due to genomic redundancy of the *RPK1*-function. Taken this into account, numerous variants of cotyledon defect variants, notably monocots, can be explained. Although it does not necessarily show how monocots evolved from dicots, our observations can be instructive to understand how a one-cotyledon phenotype could be established.

### Redundancy of RPK1 functions impacts the penetrance of the monocot phenotype

Apparently, the incomplete penetrance of the monocot phenotype of *rpk1* is due to functional redundancy. However, since *rpk1 rpk2/toad2* double mutants synergistically lead to severe morphological embryo changes, at least one of these genes exerts an additional unrelated function in the embryo. On a structural level, *RPK1* and *RPK2/TOAD2* share some conservation, especially in the kinase domain, but also have considerable differences. For instance, *RPK2* encodes a protein with 1151 amino acid residues harbouring seven LRR-domains, while RPK1 is 540 amino acids long with one LRR (supplementary material Fig. S7). Considering protein structure alone, *RPK2* looks like a duplicated copy that shares one function with *RPK1* but has potentially developed new functions. In fact, *RPK2/TOAD2* has at least one additional role in SAM organisation, together with *CLV1* and *CLV2-CRN/SOL2* (Kinoshita et al., 2010). Analysis of the evolutionary dynamics has shown that the acquisition of new functions parallel to the retention of old ones may safeguard gene duplications from being selected (Nowak et al., 1997; Cooke et al., 1997). We cannot exclude, that there is even more redundancy for the *RPK1* embryo function, as evidenced by the variable penetrance of *rpk1-7* monocot phenotype in different backgrounds. This is reminiscent of cotyledon number modifiers in *Antirrhinum* and others (Stubbe, 1963; Chandler, 2008; and references therein). Together, the data suggest that genetic and functional redundancy interferes with *RPK1*'s early function in *Arabidopsis thaliana* embryogenesis.

It should be noted, that *RPK1* also has postembryonic functions, since it is involved in ABA-related processes, especially abiotic stress tolerance (Osakabe et al., 2010). Whether there is a link between auxin-related and the ABA-related *RPK1* functions during early embryogenesis and post-embryogenesis remains to be determined. However, a previous report has pointed to the significance of ABA for early developmental stages in somatic embryogenesis of *Nicotiana plumbaginifolia* (Senger et al., 2001). In addition, Auxin and ABA are connected through FUS3 activity in late embryo development (Gazzarrini et al., 2004). There is also emerging evidence in the embryo for crosstalk of auxin with hormones like cytokinin and gibberilic acid, respectively, as can be inferred from mutants like *amp* and *ga1* (Vidaurre et al., 2007; Willige et al., 2011).

### The cotyledon anlagen are marked by the overlapping expression domains of *ANT*, *PID* and *ENP* and remain separated from the SAM in *rpk1*

The expression domains of all tested SAM genes remain essentially unchanged in *rpk1* as demonstrated by the stem cell genes *CLV1* and *3* and the more broadly expressed *STM* (Fletcher et al., 1999; Long et al., 1996). In monocot embryos the expression of *CUC2* accounts for the missing cotyledon such that the margins of the remaining cotyledon in the developing monocot embryo do not fuse, which is the basic function of *CUC* genes (Aida et al., 1999). In few cases the position of *CUC2* expression was conspicuously shifted laterally. Possibly, this marks and causes rare seedlings with retarded or no SAM. Note that at this stage it is not possible to discriminate between mono- and dicot embryos. Thus, *rpk1* effects might start at the beginning of epidermal organisation of polar PIN1. At the same time, the expression of all organ markers or key genes tested (*ANT*, *PID*, *ENP*, PIN1) is correctly present at “cotyledon positions”, regardless of whether the embryo develops one or two cotyledons. Thus, at the transition stage and beyond, the *rpk1*-embryo displays almost the wild-type spatial organisation of the molecular machinery at the embryo apex. This is in strong contrast to the defects seen in *rpk1 rpk2/toad2* double mutant embryos (Nodine et al., 2007; Nodine and Tax, 2008) and demonstrates that the *rpk1* defect is closely restricted at a cellular level to the epidermal layer.

The term anlage describes an inconspicuous cell group characterised by expression of specific genes and committed to develop into a particular tissue or organ. By contrast, a primordium originates from an anlage and is already a morphologically discernible cell group. Our results show that together the overlap of *ANT*, *PID* and *ENP* (as well as *PIN1*) expression molecularly mark the cotyledon anlage. It is best seen in monocot embryos that these genes mark cells that are prepared to but have not developed a primordium. Together with the expression of other genes (e.g. *DRN*) (Cole et al., 2009), the cotyledon anlage is thus well defined and separated from the SAM anlage, which itself is characterized by genes such as *STM* and *CLV1-3* (Long et al., 1996; Fletcher et al., 1999). It is worth mentioning that the fate-mapping experiments of Woodrick et al. showed cotyledon anlagen to initiate in a timely separated order (Woodrick et al., 2000). Since absence of *RPK1* has mild defects in comparison to the drastic alteration of PIN1 polarity in *pid enp*, this might explain why at least one cotyledon, as opposed to no cotyledon, is developed in *rpk1*.

### RPK1 acts independently from PID and ENP but *rpk1* cotyledon defects manifest downstream of PID and ENP activity

Generation of cotyledons is a complex process, which depends on numerous auxin related genes but also on the co-ordinated activity of several (transcription) factors. The combined mutations of these genes often lead, with incomplete penetrance, to abnormal cotyledons including ectopic cotyledons in the hypocotyl or cotyledons converted into root primordia (Izhaki and Bowman, 2007; Cole et al., 2009; Dhonukshe et al., 2008; Kanei et al., 2012; and references therein).

The defect in *rpk1* is different. Here, all key organisers of the apex tested are correctly expressed while defects in cell division and shape (size, oblique cell walls etc.) and PIN1 localisation are rare but detectable. These correlate with alterations of auxin maximum organization. The altered auxin maxima in turn clearly indicate a disturbed or reversed auxin-flux in the epidermis.

PIN1 is substrate of PID and other AGC kinases (Michniewicz et al., 2007; Zourelidou et al., 2009). Although this cannot be excluded, PIN1 is less likely a target of RPK1 *in vivo*, i.e. its influence on PIN1 may be indirect. Firstly, functional *RPK1* in cotyledon-less *pid enp* or *pid wag1 wag2* mutants does not even partly rescue the cotyledon defect (Treml et al., 2005; Cheng et al., 2008; Dhonukshe et al., 2010; this study). This is also corroborated by the fact that *pid enp* mutants have always a completely reverted, basal PIN1 localisation and normal cell shape (Treml et al., 2005). The defects in *rpk1* are in marked contrast to those in *pid enp*. Finally, mutations in *RPK1* affect not only PIN1 distribution but also cell division. Whether RPK1 directly or indirectly links these two processes is not clear but it has been shown that polarity of PIN proteins is a post-cytokinesis event, which results from an initially non- or bipolar arrangement (Boutté et al., 2006; Men et al., 2008). Together this shows that *RPK1* stabilises position of convergence points and auxin maxima, respectively, in an as yet unknown way on cellular level. Apparently, *RPK1* is independent of *PID* and *ENP*. However, the realisation of *rpk1* defects is only possible after *PID* and *ENP* activity. In this sense *PID* and *ENP* act before *RPK1* and further work will have to identify the ligands and targets of RPK1.

### The timely and spatially stochastic alteration of PIN1 polarity in *rpk1* explains cotyledon phenotypes

**Fig. 7.**
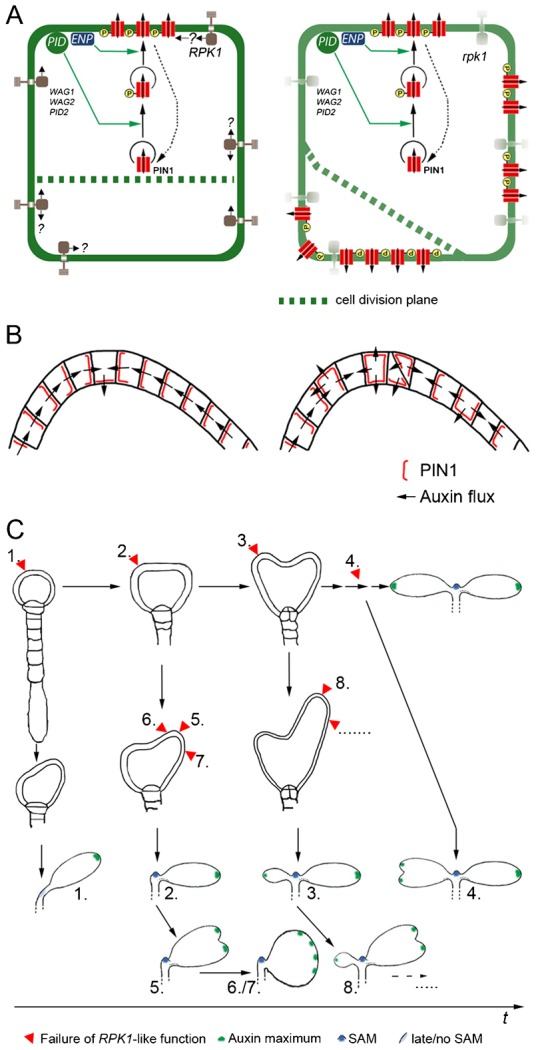
Stochastic disturbance of cell shape and PIN1 polarity in *rpk1* cells. The model illustrates the consequences of stochastic *RPK1* dysfunction during embryo development, at different levels. (A) On a cellular level: wild-type (left) compared to *rpk1* (right). RPK1 stabilises cell wall orientation and PIN1 position in the WT, in an as yet unknown fashion. In *rpk1*, in spite of a correct function of PID, ENP (and others WAG1 etc.), cell division and PIN1 localization are perturbed when the threshold of required *RPK1* function is not reached. (B) On a tissue level (the epidermal layer): wild-type (left) compared to *rpk1* (right). Patterns of cell wall and PIN1 localisation, respectively, and the deduced auxin flux are shown. (C) On an organ level: The *rpk1* effect has different outcomes depending on its manifestation along the developmental time axis. Numbers in panel C link the time and region of stochastic alteration of cell division and PIN1 polarity with the corresponding seedling morphology.

Based on the aforementioned observations and assumptions, i.e. the stochastically altered cell shape and PIN1 polarity, we have developed a model to tentatively integrate the effects of mutations in *RPK1*. In this model the generation of monocot and other cotyledon variants results from the time-point and the position of the lapse of RPK1 function (Fig. 7). This disturbance is stochastic because in every new daughter cell correct cell shape i.e. cell division and PIN1 polarity depends on whether the required threshold of RPK1-function is reached through activity of redundant genes or not (Fig. 7A,B). Thus PIN1 polarity and distribution can be compromised in more distal, i.e. at convergent points (Scarpella et al., 2006), or more lateral cells in early or late stages of development (Fig. 7C). The altered polarity will modify the size and intensity of the corresponding auxin maximum such that it will be reduced or even obliterated. Early alterations in a cotyledon anlage may retard or suppress cotyledon development completely, leading to aniso- or monocot embryos, respectively. In the extreme, this might even interfere with SAM development (1–3 in Fig. 7C). Late disturbance at or lateral to convergence points might lead to supernumerary auxin maxima and thus to partly split, fused or other cotyledon forms, for instance those with increased overall circumference (6–8 in Fig. 7C). A consequence of the stochastic effect implies that irregular dicots should also occur (4 in Fig. 7C). This is indeed the case (supplementary material Fig. S1). This model also accounts for the observation that more embryos than seedlings appeared anisocotyledonous. Since *rpk1* effects are relatively infrequent, the following normal development (due to redundancy of RPK1 function) could compensate for one or few defective cells.

Analysis of phyllotaxis and the initiation of pre-procambial elements in leaves shows clear parallels to cotyledon development but also have differences, e.g. *pid enp* mutants develop rosette leaves (Reinhardt et al., 2003; Scarpella et al., 2006; Treml et al., 2005). According to the model for adult leaf generation, leaf primordia form at sites of elevated epidermal auxin concentration initiated by newly formed convergence points. These sites in turn lead to auxin depletion in the neighbourhood, such that new primordia arise at some distance from the former (Reinhardt et al., 2003). The work of Scarpella et al. showed that venation patterns result from the balance between self-organising and genetically determined positioning of epidermal PIN1 convergence points (Scarpella et al., 2006). Convergence points are epidermal cells, which establish auxin maxima, through the accumulation of this hormone from surrounding cells with corresponding PIN1 polarity (Scarpella et al., 2006). In both cases, convergence points are evenly distributed in a field of epidermal cells due to the self-organising dynamics. Mutations in *RPK1* violate the even distribution of auxin maxima. Convergence points and auxin maxima (together with corresponding sub-epidermal pre-procambial cells) can be closely neighboured and shifted within a cotyledon or even absent resulting in larger, fused or split cotyledons. Together this shows, on a tissue level, that functional *RPK1* contributes to the stability of convergence points and auxin maxima, respectively. *RPK1*'s effect is markedly different from ABCB19/PGP19, which has been shown to stabilise PIN1 in plasma membrane micro-domains, when challenged with the detergent Triton X-100. However, PIN1 polarity is not changed in abcb19 mutants (Titapiwatanakun et al., 2009).

### Mutations in *RPK1* might extend our understanding of cotyledon evolution in angiosperms

The presented model allows considerations with respect to the evolution of cotyledons in angiosperms, which is still a matter of debate although there is a tendency to accept that monocotyly arose from dicotyly (Burger, 1998; Cooke et al., 2004; Chandler, 2008; and references therein). The presented data cannot prove the evolutionary origin of monocotyly but they provide an instructive scenario, which can help to better understand how this transition could happen.

On first approach (i.e. for *Arabidopsis*), the data show that transition from a dicot to a monocot seedling requires only one single step. At the same time the rest of the seedling body remains unaffected. Finally, a plethora of descriptions of cotyledon variants in the literature (aniso-, hetero-, syncotyl, fused, split etc. cotyledons) can be explained by the stochastic impact interfering with embryonic RPK1 and RPK1-like functions.

If one transmits these observations to other plants, this would mean that the gradual transition from dicots to monocots through intermediate cotyledon forms would not exist. Intermediate forms have, indeed, not been found. Rather, a single step would be sufficient. This has been concluded from morphological analyses of numerous species (Tillich, 1992). It is tempting to speculate that an alternative could be a progression from intermediate to elevated penetrance of cotyledon defects until reaching full penetrance. In order to stabilise monocotyly (in contrast to other cotyledon variants) a precise instead of a stochastic modulation of auxin maximum establishment in the early embryo would be required. Possibly, in *Arabidopsis* this could be achieved by impacting several (redundant) genes converting this to a complex process, which, once completed, would be difficult to revert. As a matter of fact, there seems to be no exception from monocotyly in monocots (Tillich, 1992), in contrast to the frequent occurrence of deviations from dicotyly in dicots. Probably, inclusion of additional eudicot species might support future studies addressing this question. For instance the Gesneriaceae include species, which regularly develop only one cotyledon from two pre-existing primordia (Tsukaya, 1997). Notably, this process can be experimentally manipulated by application of GA and the auxin transport inhibitor TIBA (Rosenblum and Basile, 1984). In the reclassified Hydatellaceae, which until recently belonged to the monocots (Saarela et al., 2007), a sheathing structure is interpreted as two cotyledons or at least as a case of syncotyly (Sokoloff et al., 2008). We believe that with the morphological analysis of such plants combined with modern tools of molecular biology, the test of the aforementioned scenario is within reach.

## Materials and Methods

### Plant strains and growth conditions

The Col-0 ecotype was used as wild-type reference. The homozygous *aco^FN9-3^* line, later renamed *rpk1-7*, originated from the selfing of a fast neutron mutagenized seed of Col/gl-1 background (obtained from LEHLE seeds). We used an additional *rpk1* allele (line N2995 from the Nottingham Arabidopsis Stock centre, NASC) here named *rpk1-6* (see supplementary material Table S1 for overview of *RPK1* alleles). Additional mapping lines and ecotypes, respectively, were obtained from NASC or Thomas Debener (University of Hannover). Growing and crossing of lines was essentially as previously described (Treml et al., 2005). Transgenic lines carrying *PIN1p:PIN1::GFP*, *DR5rev::GFP* and *RPK1p:RPK1::GFP* were kindly provided by J. Friml (University of Ghent) and F. Tax (University of Arizona).

### Genetic analyses, mapping and pyrosequencing

Frequency of the monocotyledonous phenotype was measured in progeny of homo- and heterozygous *rpk1-7* plants in the original or in other backgrounds. Conventional and fine mapping was carried out as described (Haberer et al., 2002). We used the visual markers *clv1*, *ap1* and *clv2*. SNP markers either derived from available L*er*/Col Sequence (Arabidopsis Genome Initiative, 2000) (TAIR) or described in Törjek et al. (Törjék et al., 2003) were assessed by pyrosequencing as described (Treml et al., 2005). Linkage analyses determined the mutation to be localized in the interval between BAC T26J14 and BAC F20P5 on chromosome 1 close to the marker *ap1* (see above and supplementary information). In a knowledge-based approach, possible candidates for *rpk1-7* were selected: *PAN*, *CRC*, *TCP15* and *RPK1*. They were subjected to further conventional or improved (hiTAIL-) PCR-analysis in wild-type and *rpk1-7* background.

### Microscopy

Semi-thin sections and whole mount analysis of embryos were carried out as previously described (Treml et al., 2005). Seedlings were processed in the same way. Photographs were taken using a ZEISS Axiophot1 microscope equipped with a Digital Nikon camera (F5SLR) and corresponding software (Nikon Camera Control Pro). Epifluorescence microscopy on the same Axiophot used a HBO50 UV/Light-source with an AHF filter system F41-017. Confocal Laser-Scanning-Microscopy was performed with an OLYMPUS FV1000/IX81 and the FluoView^TM^ software (Olympus Europa GmbH, Hamburg, Germany). Excitation of GFP probes was at 488 nm with a multi-line argon laser and fluorescence was detected using 500–550 nm slit width. One-way scan images (Kahlman frame) were obtained using an Olympus PLANPO 60× water objective.

### RT-PCR and (hiTAIL-)PCR

Plant DNA was isolated following conventional protocols. RNA isolation, reverse transcription and PCR were performed according to the supplier's instructions using a NucleoSpin®-RNA Plant (Macherey-Nagel) or PolyATract-System IV kit (Promega), respectively. For reverse transcription a TaqMan® kit (Applied Biosystems, Roche) was used. Diverse primers were selected with the aid of information from TAIR. PCR bands, generated from Col-0 and *aco^FN9-3^*/*rpk1-7* DNA as template, were sequenced through EUROFINS/MWG services.

Using an improved TAIL-PCR method (hiTAIL) (Liu and Chen, 2007) a fast neutron induced inversion was identified with specific primer combinations in *RPK1* and *At1g72250*. The following primer combinations were successfully used for hiTAIL-PCR to bridge a breakpoint: LAD1-1: ACGATGGACTCCAGAGCGGCCGC(G/C/A)N(G/C/A)NNNGGAA, AC1: ACGATGGACTCCAGAG (AC1), TRend1: CCGAATGTTCCAGCCACACCAGTTG, hiTRend2: ACGATGGACTCCAGTCCGGCC_GTGAAGATAGGAGAGAGCACGCGC, TRend3: CCTTCCACTCAATAGCAGCTTTC (TRend3). Selected primers used for PCR and partly for sequencing *RPK1* and *rpk1-7*/*At1g72250* breakpoint in Col-0 and *rpk1-7*, respectively, were: TRend4RPK1 ( = pEnd4): GAGCAGAGATCTCAGCATGAAACTG, RPKpAfw ( = pAfw): GTGAGATTCCAAAGGAGATTT, RPKpArev ( = pArev): CCTGAACCTGAGAGTTTCGTT and At1g72250 primers Afw: GCTGAAGGAGACTCAAAACATC, Cfw: ACCCTCCAAGACAAGGTAAACG and Crev: CACCACTAGGTAAAGGAGCTAG.

### In situ hybridisation analyses

In situ hybridization was essentially performed as previously published (Treml et al., 2005). Sense probes were used as controls and wild-type expression patterns for all probes were or had been previously confirmed (Treml et al., 2005; and references therein).

Kindly provided in situ probes were: p5Δ4*ANT* (*AINTEGUMENTA*, D. Smyth, University of Monash, Australia), pBSKS*CUC2* (*CUP-SHAPED COTYLEDON2*, M. Aida, Nara Institute, Japan), pBS-SKmerihb1 (*SHOOT MERISTEM-LESS*, K. Barton, Carnegie Institute Stanford, USA), pRSC1 *CLV1* (*CLAVATA1*, R. Simon, University of Düsseldorf, Germany), pNB4135 *CLV3* (*CLAVATA3*, R. Simon, University of Düsseldorf, Germany), pBSKS*PID* clone 5a (*PINOID*, S. Christensen, UCLA, USA) and pGEM-*ENP* fragment 2A (*ENHANCER OF PINOID*, a 1362 bp fragment embracing the coding for amino acids 44 to 497 cloned into pGEM®-T Easy).

Hybridizations were performed at 50°C (e.g. for the *CUC2* probe) and at 55°C (e.g. for the *ANT*, *STM* probe), respectively.

We evaluated the following numbers of embryos with the following probes. We distinguished three embryo categories: first globular and earlier stages, then cotyledon defect embryos in particular monocot embryos from transition stage onwards and finally dicot embryos from transition stage onwards (separated by slashes):

a) With *ANT*-probe: 15/17/83; b) with *CLV1*-probe: 41/8/83; c) with *CLV3*-probe: 94/20/176; d) with *CUC2*-probe: 39/14/71; e) with *ENP*-probe: 22/32/138, f) with *PID*-probe: 41/29/184 and g) with *STM*-probe: 13/22/107. Numbers are not necessarily representative for segregation; embryos were selected depending on quality and orientation.

## Supplementary Material

Supplementary Material
