# Supplementary material for: Mutations in the Arabidopsis RPK1 gene uncouple cotyledon anlagen and primordia by modulating epidermal cell shape and polarity
Source: Biol Open. 2013 Aug 22;2(11):1093–102. doi: 10.1242/bio.20135991 (PMC3828755; doi:10.1242/bio.20135991)
Supplement: Supplementary Material [file supp_2_11_1093__index.html]

Mutations in the Arabidopsis RPK1 gene uncouple cotyledon anlagen and primordia by modulating epidermal cell shape and polarity — Mutations in the Arabidopsis RPK1 gene uncouple cotyledon anlagen and primordia by modulating epidermal cell shape and polarity — Supplementary Material 

# Mutations in the *Arabidopsis RPK1* gene uncouple cotyledon anlagen and primordia by modulating epidermal cell shape and polarity

## bio.20135991 Supplementary Material

**Files in this Data Supplement:**

- Supplementary Material - Miriam Luichtl et al. doi: 10.1242/bio.20135991
