# Supplementary material for: Mutations in the Arabidopsis RPK1 gene uncouple cotyledon anlagen and primordia by modulating epidermal cell shape and polarity
Source: Biol Open. 2013 Aug 22;2(11):1093–102. doi: 10.1242/bio.20135991 (PMC3828755; doi:10.1242/bio.20135991)
Supplement: Supplementary Material [file supp_bio.20135991_bio.20135991-s1.pdf]

Biology Open

Supplementary Material

Miriam Luichtl et al. **doi: 10.1242/bio.20135991**

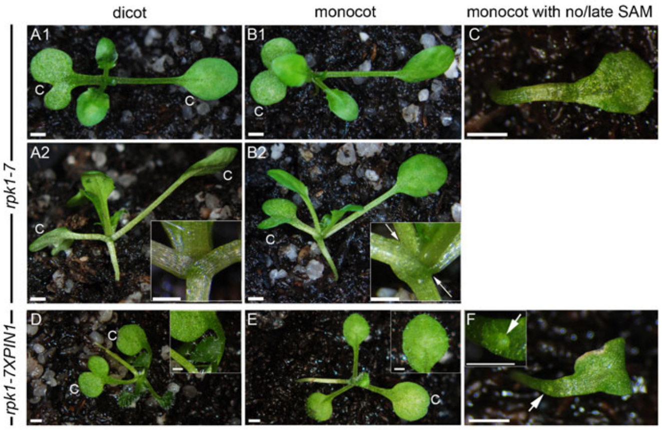

Fig. S1. Spectrum of seedling phenotypes in *rpk1-7*: cotyledon variants and SAM-less seedlings. The genetic background is indicated on the left. The first column shows irregular dicots with one cotyledon almost split in two halves. The middle column shows monocot plants where the remaining cotyledon has undergone an additional similar defect during its development. The primary leaves in *rpk1-7* background do not develop trichomes due to a *glabra1* mutation. However, dicots and monocots can be discriminated at the base of the cotyledons (compare panel A2 with panel B2 and their insets). In panel A2, primary leaves originate in between the two bases of the cotyledons whereas in panel B2 all further leaves grow on top of a swelling of the single cotyledon (separated by small arrows). Panels D and E show comparable specimens to panels A and B. However, due to the background all leaves except the cotyledons (C) now display trichomes (insets). Right column with a SAM-less monocot (in panel C) and one, which has generated a late leaf primordium (in panel F). A "C" indicates a cotyledon; arrows point to the base of cotyledons and a leaf primodium (in panel F), respectively. Scale bars: 1 mm, 500 µm (insets).

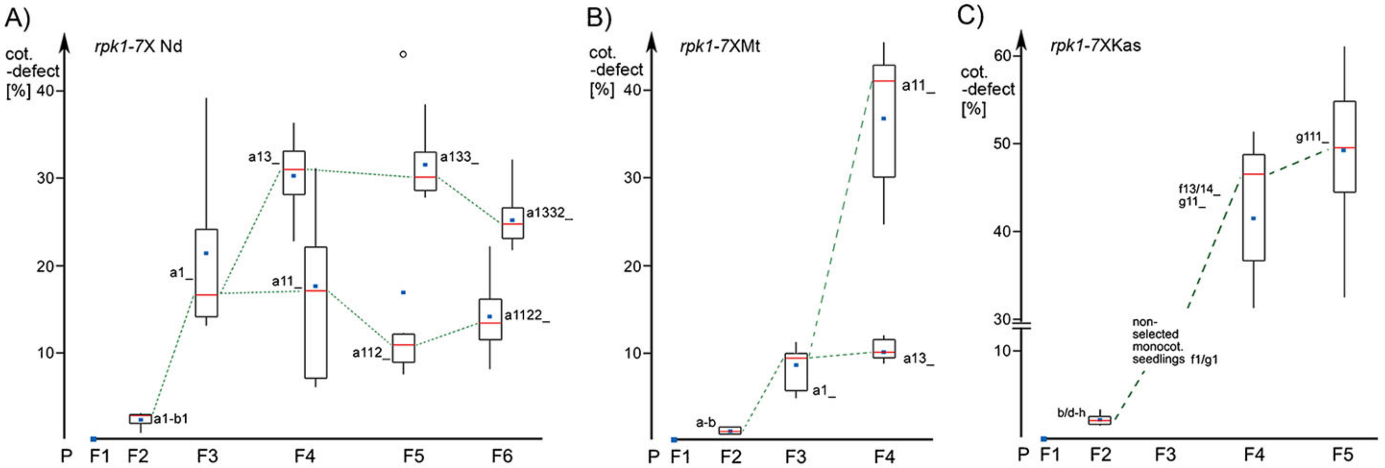

Fig. S2. Boxplot representation of *rpk1-7* cot.-defect frequency in other ecotype backgrounds. *rpk1-7* was crossed with ecotypes Nd (A), Mt (B) and Kas (C). F1 had no monocot seedlings and was selfed. In subsequent generations (from F2 onwards) several monocot seedlings were grown, selfed and their pedigree assessed with respect to monocot frequency. Some monocot seedlings of lines with high (or low as in Nd-cross) frequency of cot.-defect seedlings were grown and selected again. Note that in all three cases, especially in the cross with Kas, increasing frequency was accompanied by increasing numbers of monocot seedlings with abnormally lobed cotyledons. The F2 had an expected low frequency because the F1 produces 25% *rpk1-7* homozygotes, which give ca. 2.5% monocots if the penetrance for monocot seedling is 10%. Blue square: average, red line: median, circle: outlier, box: position of lower and upper quartile, respectively, black vertical line: smallest and largest observations except outliers. Letters with numbers indicate the parent line whose pedigree was taken for further rounds of selection.

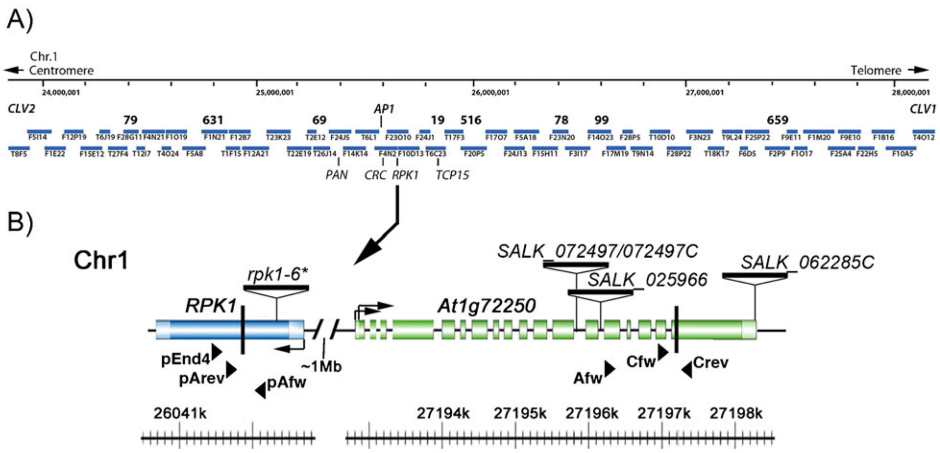

Fig. S3. Overview of the *RPK1* chromosomal region and used *rpk1* and *At1g72250* alleles. (A) Contig of the *RPK1*-region. The scheme shows the contig taken from the AGI information (TAIR) spanning the region between *CLV2* and *CLV1* on bottom of *Arabidopsis* Chromosome 1. Tested genes representing possible *aco*$^{FN\text{-}9\text{-}3}$ mutant candidates are indicated beneath the contig. Numbers in bold represent positions of SNPs used for fine mapping (SNP sequences are available on request). (B) Schematic representation of the alleles used in this work. Position of the used T-DNA insertion alleles. Position of *rpk1-6* was taken from NASC. Positions of *At1g72250* alleles (SALK_072497/C, SALK_025966, SALK_062285C) were determined by sequencing.

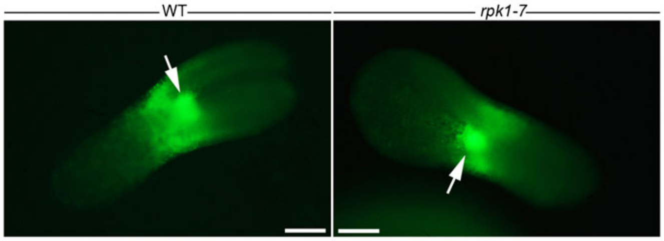

Fig. S4. *CLV1p:CLV1::eGFP* in dicot and monocot *rpk1-7* embryos. Left: GFP fluorescence in wild-type dicot embryo. Right: the same in a monocot *rpk1-7* embryo. Strong signal intensity is seen in the SAM-region (arrows). Note that the brightness of the figure has been strongly enhanced such that the dicot is distinguishable from the monocot and that this construct additionally exhibits some fluorescence at the hypocotyl flanks to both sides of the SAM. Probably the promoter fragment in the construct is incomplete. Scale bars: 10 µm.

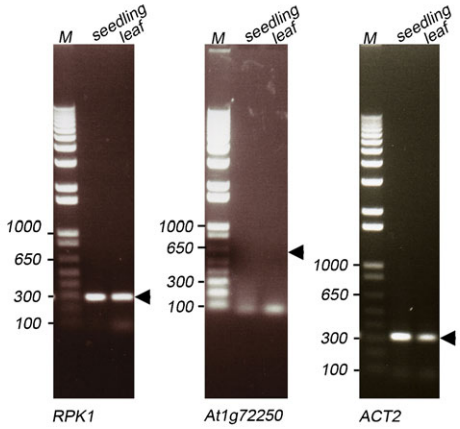

Fig. S5. Expression of *RPK1*, *At1g72250* and *ACT2* in *enp pid* seedlings and leaves. Total RNA of seedlings and leaves from *pid enp* double mutants was taken for RT-PCR of the three genes. Arrowheads indicate position of expected (cDNA-) band with the primers used.

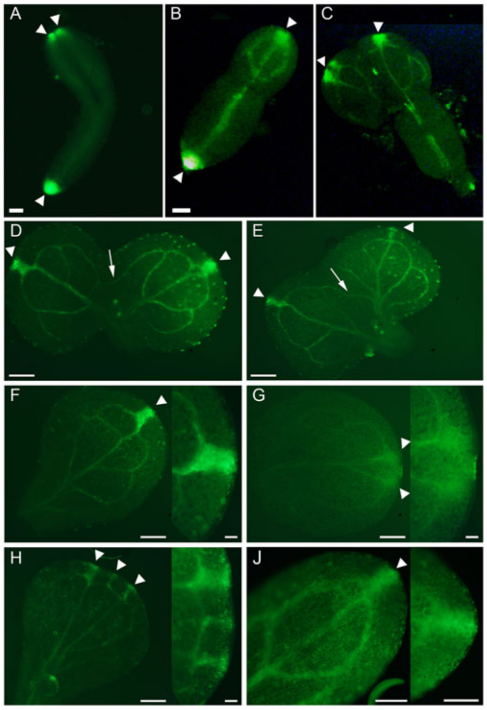

Fig. S6. Variable auxin maxima in wild-type and *rpk1-7* embryos and seedlings. (A) Wild-type dicot embryo. (B) *rpk1-7* monocot embryo. (C) *rpk1-7* monocot embryo with enlarged cotyledon and two auxin maxima (root part lost during isolation). (D–H) Seedlings displaying irregular auxin maxima. The arrows in panels D and E point to vascular connections between the larger parts of the abnormal cotyledon. They show that the cotyledon did not arise by fusion of two embryonic cotyledons along their margins. (J) Wild-type seedling. Arrowheads point to auxin maxima. Insets show magnifications. Scale bars: 30 µm (A–C), 100 µm (D–J).

Biology Open

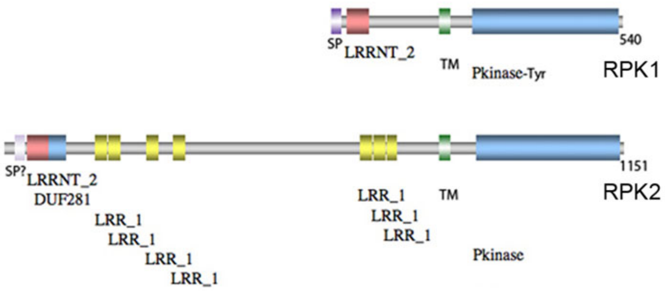

Fig. S7. Structure of *RPK1* and *RPK2/TOAD2*. Schemes and data from "The Plant Proteome Database" at Cornell University for *RPK1*: http://cbsusrv04.tc.cornell.edu/users/ppdb_domain/hmmpfam.aspx?id=5769&eval=1 and *RPK2/TOAD2*: http://cbsusrv04.tc.cornell.edu/users/ppdb_domain/hmmpfam.aspx?id=11514&eval=1. Pkinase: Protein kinase domain, TM: Trans-membrane domain, LRR: Leucine-rich repeat domain, LRRNT: Leucine-rich repeat N-terminal domain, DUF: Domain of unknown function.

Table S1. Overview of existing *RPK1* mutant alleles.

Allele number	Mutation	Strength	Background	Publication
rpk1-1	+504 bp from ATG T-DNA insertion	Strong n. m.	WS Wisconsin *Arabidopsis* knockout facility	Osakabe et al., 2005
*rpk1-1**	+502 bp from ATG T-DNA insertion; very likely identical to *rpk1-1*	Strong 6.8% cot defects.	WS Wisconsin *Arabidopsis* knockout facility Nodine et al., 2007; backcrossed to Col.	Nodine et al., 2007; Nodine and Tax, 2008
rpk1-2	+989 bp from ATG T-DNA insertion	Germination and stomata: weaker than *rpk1-1*; n. m.	WS Wisconsin *Arabidopsis* knockout facility	Osakabe et al., 2005
rpk1-3	In the kinase domain; DS insertion	Senescence: strong; n. m.	Unknown Cold Spring Harbor collection Sundaresan et al., 1995	Lee et al., 2011
rpk1-4	In the kinase domain close to *rpk1-3* DS insertion	Senescence: comparable to *rpk1-3*; n. m.	Unknown Cold Spring Harbor collection Sundaresan et al., 1995	Lee et al., 2011
rpk1-5	Point mutation (W140*)	Weaker than *rpk1-1**, 5.4% cot defects	Col *er105* is the 3rd backcross of Col *er105* fast neutron mutant (Torii et al., 1996) Arabidopsis TILLING Project (ATP) facility (Till et al., 2003) Nodine et al., 2007 backcrossed to Col.	Nodine et al., 2007
rpk1-6	357 bp from ATG, T-DNA Insertion; NASC by F. Tax.	Comparable to *rpk1-1**, 7.15% cot defects	WS-2 Wisconsin *Arabidopsis* knockout facility	NASC, this study
rpk1-7	Fast neutron induced inversion with breakpoints in *RPK1* and *At1g72250*	Stronger than *rpk1-1**, 10.5% cot defects	Col with *gl-1* background	This study

n. m.: cotyledon defects were not analysed.

Table S2. Frequencies of embryos diverging from wild-type at different stages.

Embryo stage*	Quadrant	Octant	16cell	Globular	Transition	Early heart	Mid heart	Late heart	Torpedo**
Plant no. 1	0% (*n*=1)	0% (*n*=4)	0% (*n*=3)	30% (*n*=43)	0% (*n*=2)	41% (*n*=17)	27% (*n*=22)	25% (*n*=16)	23% (n=18)
no. 2	– (*n*=0)	0% (*n*=7)	0% (*n*=13)	41% (*n*=68)	25% (*n*=8)	37% (*n*=35)	50% (*n*=12)	30% (*n*=20)	29% (n=7)
no. 3	0% (*n*=1)	0% (*n*=7)	0% (*n*=12)	19% (*n*=65)	25% (*n*=16)	25% (*n*=32)	20% (*n*=45)	28% (*n*=32)	– (n=0)
no. 4	0% (*n*=2)	0% (*n*=13)	0% (*n*=14)	55% (*n*=66)	50% (*n*=12)	27% (*n*=41)	46% (*n*=11)	– (*n*=0)	– (n=0)
no. 5	– (*n*=0)	0% (*n*=8)	0% (*n*=14)	64% (*n*=55)	50% (*n*=2)	50% (*n*=12)	33% (*n*=3)	29% (*n*=7)	0% (n=4)

*Plants numbered 1 and 2 were of Col-0 background. Plants numbered 3–5 were of mixed Col-0/L*er* background.
**Only early torpedo stages counted.

Biology Open
